# Predictors of adult outcomes in clinically- and legally-ascertained youth with externalizing problems

**DOI:** 10.1371/journal.pone.0206442

**Published:** 2018-11-01

**Authors:** Richard Border, Robin P. Corley, Sandra A. Brown, John K. Hewitt, Christian J. Hopfer, Michael C. Stallings, Tamara L. Wall, Susan E. Young, Soo Hyun Rhee

**Affiliations:** 1 Institute for Behavioral Genetics, University of Colorado Boulder, Boulder, CO, United States of America; 2 Department of Psychology and Neuroscience, University of Colorado Boulder, Boulder, CO, United States of America; 3 Department of Applied Mathematics, University of Colorado Boulder, Boulder, CO, United States of America; 4 Department of Psychiatry, University of California, San Diego, La Jolla, CA, United States of America; 5 Department of Psychiatry, Anschutz Medical Campus, University of Colorado Denver, Aurora, CO, United States of America; University of Sao Paulo Medical School, BRAZIL

## Abstract

Externalizing problems (EP), including rule-breaking, aggression, and criminal involvement, are highly prevalent during adolescence, but the adult outcomes of adolescents exhibiting EP are characterized by heterogeneity. Although many youths’ EP subside after adolescence, others’ persists into adulthood. Characterizing the development of severe EP is essential to prevention and intervention efforts. Multiple predictors of adult antisocial personality disorder (ASPD) and legal outcomes of a large sample (*N* = 1205) of clinically- or legally-ascertained adolescents (ages 12–19 years) with severe EP were examined. Many psychosocial predictors hypothesized to predict persistence of EP demonstrated zero-order associations with adult outcomes, but accounted for little *unique* variation after accounting for baseline conduct disorder symptoms (CD) and demographic factors. Baseline measures of intelligence, which explained independent variation in legal outcomes, provided the only consistent exception to this pattern, though future work is needed to parse these effects from those of socioeconomic factors. CD severity during adolescence is a parsimonious index of liability for persistence of EP into adulthood that explains outcome variance above and beyond all other demographic and psychosocial predictors in this sample.

## Introduction

Externalizing problems, including legal involvement, substance use problems, and conduct disorder (CD) symptoms, are common during adolescence and frequently precede negative psychosocial and legal sequelae [1]. Despite the high prevalence of ASB during adolescence—e.g., the estimated lifetime prevalence of CD is 9.5% [2]—the adult outcomes of adolescents with ASB are heterogeneous; many youth desist from ASB during the transition into adulthood, whereas others persist [3]. Researchers have defined discrete groups of youth with ASB based on their developmental trajectories (e.g., persisters versus desisters) and compared their psychosocial profiles to identify potential risk factors. These studies have typically examined constellations of risk factors exerting reciprocal influences across development, wherein distinct vulnerabilities are rarely thought to operate in mutual isolation [1,4]. Though many candidate risk factors have been identified in this fashion, this approach is not without its weaknesses. First, trajectory-based categorization of individuals requires information typically unavailable to prevention and intervention efforts (e.g., age-of-onset, repeated measures across development). Second, the fact that individuals with different trajectories exhibit mean differences in a variety of psychosocial factors does not imply that measures of said factors during adolescence will explain *independent* variation in adult outcomes. Thus, whether previously identified correlates of persistence consistently account for outcome variation in adult in beyond baseline CD and demographic factors—information crucial to decisions of which traits to measure in intervention/prevention settings where time and resources are limited—remains unclear. To address these gaps, the present study examined the adult outcomes of clinically- and legally-ascertained adolescents with CD symptoms and substance use problems [SUP], investigating the effects of many previously-identified predictors of persistence (e.g., verbal deficits, SUP severity, maternal ASPD, familial cohesion and conflict, and perceived peer deviance) beyond those of baseline CD and demographic factors. Analyses aimed to highlight predictors of persistence at a time point particularly relevant to intervention efforts (i.e., clinical and/or legal ascertainment).

### Heterogeneity in the persistence of ASB

Moffitt advanced the highly influential “developmental taxonomic theory” of ASB outlining two distinct behavioral trajectories: adolescent-limited (AL) and life-course-persistent (LCP) ASB [3]. Individuals with AL ASB temporarily exhibit ASB normative within the context of adolescence. In contrast, LCP youth are characterized by unique neurocognitive profiles that emerge in early childhood and interact with negative environmental influences (e.g., parental neglect) to increase the likelihood that their behaviors will persist into adulthood.

Numerous investigations have reported findings congruent with Moffitt's distinction between EP limited to adolescence versus that which persists into adulthood, though the exact number of groups with distinct trajectories (e.g., adult-onset, childhood-limited, etc.) is highly variable across studies [5]. Early longitudinal research identified AL/LCP groupings through theory-driven categorization procedures based on individual ASB trajectories and, more recently, studies employing latent class analyses to classify individuals by trajectory have supported this distinction [5,6]. However, whether or not the results from methods that categorize individual trajectories into distinct groupings (i.e., AL vs. LCP) permit investigators to determine if qualitative differences exist between groups is a point of controversy [7,8]. Additionally, many adolescent-onset ASB youth frequently continue offending into adulthood and evidence legal and psychosocial outcomes analogous to those of their childhood-onset counterparts [9]. Though these trajectory-based investigations have identified numerous putative risk factors for persistent ASB, their results do not directly inform choices of which traits are likely to be informative in samples of youth already facing correctional action for whom no longitudinal data are available.

### Correlates of persistence

Comparisons of AL and LCP ASB suggest a variety of psychosocial correlates associated with persistence. Below, we review those available in the current investigation, for the purpose of identifying any predictors of persistence beyond baseline ASB and demographic measures.

#### Demographic factors

Male gender is a robust predictor of ASB across the lifespan, and females with ASB are more likely to be classified as AL [10]. Age provides developmental context for evaluating the severity of EP (e.g., theft of alcohol at age 12 years may indicate greater severity than the same transgression at age 18 years). Verbal deficits.

Antisocial youth often exhibit verbal IQ deficits [11–14], and LCP individuals may have greater deficits in verbal capabilities than AL individuals through a variety of pathways (e.g., academic failure, poor social capabilities) [15]. However, the association between earlier verbal deficits and EP in adulthood is characterized by mixed findings [12,14,16,17], with some work suggesting that the degree to which verbal deficits predict the persistence of CD symptoms depends on parental antisocial personality disorder (ASPD) diagnosis (i.e., the presence of a parent with ASPD negates the potential protective effects of higher-than-average verbal capabilities) [18]. It is currently unknown whether verbal deficits account for independent variation in EP during adulthood after adjusting for baseline severity of EP and demographic factors in adolescence, and the predictive utility of verbal deficits in clinical settings remains unclear.

#### Externalizing problems in adolescence

CD and SUP during adolescence are both known to predict future conduct problems and criminal activity [4,19]. However, it is difficult to distinguish between the repeated effects of underlying traits versus the cascading effects of repeated offenses (e.g., one offense may lead to school expulsion, which may place the individual at increased risk for repeated offenses). Further, the extent to which SUP accounts for unique variation in adult EP beyond adolescent EP is unclear; commonly employed trajectory-based models of EP have suggested that both AL and LCP youth demonstrate elevated levels of SUP [4], but do not directly speak to the independent predictive utility of either construct [7,8]. Additionally, some research has suggested that independently examining qualitatively distinct CD symptom clusters, i.e. aggressive symptoms versus non-aggressive symptoms, may improve our ability to distinguish between persistent and adolescent-limited EP [20,21], though evidence as to whether these symptom clusters reflect distinct genetic and environmental influences is mixed [22,23].

#### Familial ASB and family environment

A variety of familial factors including family conflict, family cohesion, and parental antisocial behavior have been associated with EP during adulthood [18,24–28]. The importance of patterns of familial interaction is further supported by evidence for the efficacy of family- and parenting-oriented treatments for childhood disruptive behavior disorders [29–31]. Additionally, evidence from twin research indicates that individual differences in CD and SUP across childhood and adolescence reflect substantial genetic underpinnings [32,33]. In the present study, measures of paternal ASPD were unavailable, so we restricted our focus to maternal ASPD. Though the relative importance of maternal versus paternal psychiatric disorders in predicting offspring outcomes is unknown [34], previous research has suggested that maternal antisocial behavior predicts future child disruptive behavior disorder outcomes and related outcomes [21,35]. Due to the multiple potential pathways linking familial variables and EP (i.e., transmission through latent genetic vulnerabilities versus transmission via environmental consequences), however, the predictive utility of familial factors, particularly among adolescents with EP, remains unclear.

#### Deviant peer influence

Association with antisocial peers has been hypothesized to explain ASB in AL youth and previous work has suggested that peer deviance is associated with greater ASB during early and late adolescence [1,4,6,17]. However, whether exposure to deviant peers further predicts persistence after accounting for EP during adolescence is unclear, and gene–environment correlation may also contribute to the putatively environmental consequences of affiliation with deviant peers [36]. Beyond elucidating the predictive utility of deviant peer affiliation, further examination of this association is crucial, as clinical and legal interventions may place individuals in further contact with deviant peers [37].

### The present study

The factors explaining unique variance in the adult outcomes of clinically- and legally-ascertained adolescents beyond demographic factors and initial EP remain unclear. Many existing longitudinal studies following adolescents with EP through the transition into adulthood have focused on group comparisons with respect to discrete developmental trajectories. Though this approach has informed the examination of potential correlates and causes of EP persistence, it has two major shortcomings in its ability to inform intervention efforts in adolescence, when individuals are most likely to be ascertained. First, it requires longitudinal information not typically available to clinical and legal institutions. Second, it does not speak to the utility of measuring specific risk factors in high-risk youth ascertained as adolescents. In this context, the present study examined the unique contributions of all available psychosocial predictors (i.e., verbal deficits, CD, SUP, maternal ASPD, family environment, and deviant peer influence) beyond baseline demographic factors and CD symptoms to adult ASPD and involvement with the criminal justice system in a longitudinal sample of 1205 clinically- and legally-ascertained youth.

## Methods

### Participants

The current longitudinal, multi-site sample is composed of youth with severe EP. Between 1993 and 2007, 1517 adolescents were recruited from residential and outpatient treatment facilities for substance abuse and delinquency, criminal justice records, schools for youth with behavior problems, and alcohol and drug treatment programs [38–40].

Participants from the *1993–1997 Denver Clinical Sample* (*n* = 244) consisted of males recruited from Denver, Colorado area residential facilities for substance abuse and delinquency; Participants from the *1997–2002 Denver Clinical Sample* (*n* = 362) sample were recruited from residential and outpatient treatment facilities for substance abuse and delinquency; Participants from the *2001–2006 Denver Clinical Sample* (*n* = 363) were recruited from outpatient substance abuse treatment programs; Participants from the *Denver Adjudicated* sample were identified through Colorado criminal justice records (*n* = 302); Participants from the *San Diego Sample* (*n* = 246) sample were recruited from schools for youth with behavior problems and alcohol and drug treatment programs in San Diego, California. With respect to the 1993–1997 and the 1997–2002 Denver clinical samples, participants for whom a first degree relative agreed to participate in the assessments were targeted for follow-up. With respect to the remaining samples, participants who displayed at least one CD symptom or at least one non-tobacco SUD symptom at initial assessment were targeted for follow-up. All targeted participants (*N* = 1205) completed between one and two follow-up assessments, each occurring approximately five years apart, with the most recent follow-up assessment occurring at an average of 9.21 years (*SD = 3*.*41*) subsequent to baseline assessment. Some participants participated in two follow-up assessments of ASPD symptoms, and repeated measures were utilized when available. See Table 1 for detailed descriptions of the five samples and characteristics of participants targeted for follow-up.

**Table 1:** Sample characteristics and descriptive statistics.

	Denver Clinical 1993–1997	Denver Clinical 1997–2002	Denver Clinical 2001–2006	Denver Adjudicated	San Diego	Entire Sample
*N baseline*	126	268	362	207	242	1205
*N follow-up*	95	234	228	133	205	895
*Age at baseline*	15.85 [1.35]	15.71 [1.24]	16.18 [1.05]	17.18 [1.30]	16.61 [1.11]	16.30 [1.28]
*Age at follow-up*	28.23 [2.66]	27.07 [2.83]	23.65 [2.76]	24.81 [3.59]	25.19 [3.40]	25.53 [3.42]
*Sex (% female)*	0.00%	13.43%	15.75%	24.64%	36.78%	19.34%
*Race/ethnicity*:						
*% African-Amer*. *(non-Latino)*	4.76%	7.46%	9.12%	5.80%	9.50%	7.80%
*% Caucasian (non-Latino)*	53.17%	55.97%	56.08%	70.53%	34.71%	53.94%
*% Latino*	33.33%	30.22%	21.27%	10.63%	39.67%	26.39%
*Vocabulary*	-0.23 [0.85]	-0.08 [1.02]	0.02 [0.88]	0.35 [1.06]	-0.05 [0.99]	0.03 [0.98]
*Block design*	0.18 [0.96]	-0.13 [1.14]	0.05 [0.88]	0.08 [1000]	0.09 [0.98]	0.03 [0.99]
*Family cohesion*	—	0.22 [0.99]	0.05 [0.95]	-0.17 [0.94]	-0.29 [1.00]	-0.05 [1.00]
*Family conflict*	—	-0.23 [0.97]	-0.13 [0.96]	0.06 [0.97]	0.35 [0.93]	0.03 [0.98]
*Substance vulnerability*	0.18 [0.99]	0.01 [0.86]	0.23 [0.93]	-0.26 [0.88]	0.20 [0.99]	0.09 [0.94]
*Perceived peer deviance*	0.30 [0.86]	-0.08 [1.00]	—	-0.39 [0.88]	—	-0.01 [0.97]
*Maternal ASPD*	1.39 [1.43]	2.18 [1.74]	—	2.10 [2.00]	—	5.51 [2.79]
*Baseline CD*	6.52 [2.09]	5.88 [2.75]	5.72 [3.08]	4.44 [2.55]	5.17 [2.54]	1.96 [1.78]
*Baseline CD*: *aggressive sx*.	1.81 [1.35]	1.88 [1.57]	1.85 [1.68]	1.37 [1.54]	1.69 [1.54]	1.74 [1.58]
*Baseline CD*: *non-aggressive sx*.	4.71 [1.23]	3.99 [1.64]	3.87 [1.77]	3.07 [1.46]	3.48 [1.47]	3.77 [1.64]
*Lifetime ASPD*	5.16 [1.70]	4.64 [1.90]	3.92 [1.97]	3.41 [2.00]	3.68 [1.88]	4.11 [1.99]
*Past year ASPD*	1.29 [1.19]	1.45 [1.32]	1.71 [1.67]	1.34 [1.67]	1.78 [1.64]	1.56 [1.54]
*Recent legal involvement*	61.54%	41.52%	25.44%	57.89%	73.04%	51.94%
*Arrest after 18*^*th*^ *birthday*^†^	96.83%	85.95%	77.17%	48.35%	41.27%	68.41%
*Attrition*	24.60%	12.69%	37.02%	35.75%	15.29%	25.73%

*Note*. Em dashes indicate that a measure was not administered to participants from the given sample. With the exception of sample sizes, dichotomous variables, and age ranges, the mean, [standard deviation] is presented for each factor in each sample. Vocabulary and block design scores were standardized with respect to national norms with a mean of 10 and a standard deviation of 3.

^†^Arrest after 18^th^ birthday only presented for individuals under 18 years of age at baseline.

Inclusion criteria for all studies were comprised of the following: (a) absence of psychosis, intellectual disability, and imminent danger to self or others; and (b) absence of physical illness or current intoxication precluding participation in treatment or evaluation. Written consent from parent or guardian and assent from the participant was obtained for all participants under age 18 years and written consent was obtained for each adult. Participants received monetary compensation. All study procedures received institutional review board approval prior to data collection by the review boards of the University of Colorado Boulder, the University of Colorado Denver, and the University of California, San Diego.

### Measures

#### Intelligence

Baseline verbal and performance capabilities were assessed using the vocabulary and block design subtests from the Wechsler Abbreviated Scale Intelligence [41,42], the Wechsler Adult Intelligence Scale [43], or the Wechsler Intelligence Scale for Children [44], depending on the sample and the version available at the time. Scaled scores relative to national norms were used.

#### Family environment

Family environment was assessed using a shortened and simplified version of the Family Environmental Scale (FES) [45,46]. The present study examined the family conflict and family cohesion subscales.

#### Substance abuse/dependence vulnerability

The Composite International Diagnostic Interview-Substance Abuse Module (CIDI-SAM) [47] provides symptom counts and diagnoses of abuse and dependence for 11 categories of substances according to Diagnostic and Statistical Manual of Mental Disorders, fourth edition (DSM-IV) guidelines [48]. A composite substance abuse/dependence vulnerability index was generated by dividing the total number of abuse/dependence and symptoms by the number of substances tried and can be interpreted as average abuse and dependence symptoms per substance [49].

#### Perceived peer deviance

Perceived peer delinquency was measured with the Exposure to Delinquent Peers Measure [50]. Participants indicated their perceptions of the proportion of friends who had participated in delinquent behaviors.

#### CD and antisocial personality disorder (ASPD)

Baseline lifetime CD criteria count was assessed using either the Diagnostic Interview Schedule for Children (DISC) version 2.1 or the DISC-IV, depending on sample [51,52]. Although two additional CD symptoms (a 14^th^ and 15^th^) were added in the fourth iteration of the DSM, correlations between DSM-III and DSM-IV-based symptom counts among participants interviewed with the DISC-IV approached unity (*r* = 0.978; *r* = 0.963 and *r* = 0.972 for aggressive symptoms and non-aggressive symptom clusters, respectively). Baseline maternal lifetime ASPD criteria count was assessed using the Diagnostic Interview Schedule (DIS) version III-R or version IV [53,54]. Both past-year and lifetime ASPD criteria count were measured using the DIS-IV at follow-up. Models examining these outcomes controlled for participant age at follow-up.

#### Legal outcomes

Arrest after 18^th^ birthday was assessed using a single self-report item from the DIS-IV, and all models examining this outcome were adjusted for participant age at follow-up. Participants who were 18 or older at baseline were excluded from analysis. Recent legal involvement was also obtained; in a living arrangements interview, participants indicated whether they had spent on any time parole, on probation, or incarcerated during each of the previous three to five years (since last assessment). As a small subset of participants only had data available for the past three or four years, the number of years of data availability was included as a covariate in all relevant analyses.

### Data analysis

Data manipulation was performed in the R programming environment for GNU/Linux [55]. Analyses were conducted in Bayesian framework utilizing Hamiltonian Markov Chain Monte Carlo sampling with weakly informative priors via the probabilistic programming language Stan and its interfaces with R [56–58].

Bivariate associations (Pearson, polyserial, and polychoric correlations) between all variables were estimated using the *polycor* package [59] (Table 2). Baseline CD symptoms, demographic variables, and outcome variables were separately modeled in the context of Bayesian generalized linear mixed models (GLMMs), with the choice of distributional family and link function based on their empirical distributions. For each outcome, we first estimated a *compact model*, which included age at baseline, age(s) at follow-up(s), ethnicity, sex, and baseline CD as predictors. We then estimated separate *augmented* models for each additional psychosocial predictor by re-estimating the compact model while including the additional predictor. This procedure was chosen for two reasons. First, as not all samples were administered every measure (Table 1), including all additional psychosocial predictors would have dramatically reduced effective sample size. Second, each augmented model reflects the additional unique information gained by considering an additional predictor on top of baseline CD symptoms and demographic figures, thus specifically informing decisions regarding the utility of including additional measures in assessments in a prevention context.

**Table 2:** Bivariate associations.

	*Age at baseline*	*Age at follow-up*	*Sex*	*Vocabulary*	*Block design*	*Family cohesion*	*Family conflict*	*Substance vuln*.	*Per*. *peer del*.	*Maternal ASPD*	*Baseline CD*	*Agg*. *CD Sx*	*Non-agg*. *CD Sx*
*Age at follow-up*	0.201**												
*Sex*	-0.003	0.018											
*Vocabulary*	0.112**	-0.014	-0.038										
*Block design*	0.132**	0.017	0.112*	0.342**									
*Family cohesion*	0.009	0.060	0.101*	0.020	-0.029								
*Family conflict*	0.041	-0.056	-0.194**	0.144**	0.100*	-0.478**							
*Substance vulnerability*	0.048	0.019	0.023	0.004	0.049	-0.014	0.012						
*Per*. *peer delinquency*	-0.075	0.040	0.473**	-0.073	0.007	-0.147*	0.149*	0.193**					
*Maternal ASPD*	-0.104*	-0.067	-0.170*	-0.177**	-0.164*	0.050	0.020	-0.125*	-0.129*				
*Baseline CD*	-0.110**	0.022	0.255**	-0.012	0.039	-0.072*	0.098*	0.296**	0.478**	-0.038			
*Baseline CD*: *aggressive sx*.	-0.047	0.008	0.264**	-0.029	0.008	-0.059	0.120**	0.234**	0.432**	0.004	0.859**		
*Baseline CD*: *non-aggressive sx*.	-0.140**	0.030	0.183**	0.008	0.059	-0.065	0.049	0.278**	0.384**	-0.068	0.870**	0.494**	
*Lifetime ASPD*	-0.085*	0.098*	0.368**	-0.065	-0.010	-0.011	0.020	0.077*	0.194**	0.012	0.302**	0.256**	0.263**
*Past year ASPD*	-0.028	-0.029	0.169**	-0.020	0.056	-0.040	0.065	0.042	0.009	0.017	0.134**	0.146**	0.087*
*Recent legal involvement*	-0.148*	-0.139*	0.442**	-0.147*	-0.099*	0.116*	-0.160*	0.084	0.176*	0.169*	0.141*	0.121*	0.123*
*Arrest after 18*^*th*^ *birthday*^†^	-0.153*	0.103*	0.543**	-0.149*	-0.063	0.124*	-0.197**	0.008	0.193*	-0.004	0.231**	0.182**	0.214**
*Attrition*	-0.026	—	0.066	-0.147**	-0.064	-0.116*	0.042	-0.049	-0.009	0.082	0.036	0.049	0.015

*Note*. Unadjusted bivariate associations between measured variables. Coefficients represent Pearson, polyserial, or polychoric correlations. Age at follow-up, lifetime ASPD, and past year measures are presented for most recent follow-up assessment, though principle analyses utilize data from multiple time points where available.

*p < .05

**p < .001

^†^Arrest after 18^th^ birthday only presented for individuals under 18 years of age at baseline.

A mixed models random-intercepts framework was implemented to account for dependence among observations nested within samples, and for repeated-measures variables, within individuals. Baseline CD and lifetime ASPD were modeled with linear mixed effects regression [60], past year ASPD was modeled with negative-binomial mixed effects regression [61], and binary outcomes (arrest after 18^th^ birthday, attrition, and legal involvement during the past five years) were modeled with logistic mixed effects regression [60]. Each outcome was first regressed on sex, age at baseline, age(s) at follow-up(s), ethnicity, and baseline CD symptoms (compact models, Table 3). Additional psychosocial variables were then included one at a time to assess their independent contributions beyond baseline CD and demographic variables (augmented models, Table 3).

**Table 3:** Generalized linear mixed model results: antisocial behavior outcomes.

**Lifetime ASPD**		***β***	**95% CI**	***SE***	Observations	Individuals	Sample
Compact model							
	*Baseline CD*	0.462*	0.342–0.585	0.063	1024	885	5
	*Age baseline*	0.043	-0.062–0.154	0.055	1024	885	5
	*Age follow-up*	-0.032	-0.075–0.011	0.022	1024	885	5
	*Sex (male vs*. *female)*	0.919*	0.609–1.221	0.156	1024	885	5
	*Ethnicity*^†^	—	—	—	1024	885	5
Augmented models							
	*Substance vulnerability*	0.020	-0.103–0.147	0.065	1024	885	5
	*Block design*	0.041	-0.087–0.178	0.068	898	778	5
	*Vocabulary*	-0.031	-0.168–0.105	0.070	898	778	5
	*Family cohesion*	-0.068	-0.212–0.075	0.072	755	616	4
	*Family conflict*	0.110	-0.036–0.258	0.075	754	615	4
	*Maternal ASPD*	0.033	-0.146–0.225	0.095	470	355	3
	*Perceived peer deviance*	0.182	-0.040–0.403	0.112	437	322	3
**Past year ASPD**		**e**^*β*^	**95% CI**	***SE***	Observations	Individuals	Samples
Compact model							
	*Baseline CD*	1.126*	1.066–1.190	0.028	1024	885	5
	*Age baseline*	1.008	0.960–1.060	0.025	1024	885	5
	*Age follow-up*	0.983	0.961–1.006	0.012	1024	885	5
	*Sex (male vs*. *female)*	1.263*	1.091–1.462	0.074	1024	885	5
	*Ethnicity*^†^	—	—	—	1024	885	5
Augmented models							
	*Substance vulnerability*	0.987	0.931–1.046	0.030	1024	885	5
	*Block design*	1.059	0.996–1.125	0.031	898	778	5
	*Vocabulary*	1.008	0.945–1.074	0.032	898	778	5
	*Family cohesion*	0.959	0.900–1.021	0.032	755	616	4
	*Family conflict*	1.059	0.992–1.127	0.033	754	615	4
	*Maternal ASPD*	1.015	0.931–1.106	0.044	470	355	3
	*Perceived peer deviance*	0.960	0.868–1.063	0.052	437	322	3
**Baseline CD**		*β*	**95% CI**	***SE***	Observations	Individuals	Samples
Compact model							
	*Age baseline*	-0.054	-0.117–0.009	0.032	1205	1205	5
	*Sex (male vs*. *female)*	0.368*	0.222–0.512	0.073	1205	1205	5
	*Ethnicity*^†^	—	—	—	1205	1205	5
Augmented models							
	*Substance vulnerability*	0.292*	0.239–0.345	0.027	1205	1205	5
	*Block design*	0.047	-0.016–0.110	0.033	1059	1059	5
	*Vocabulary*	0.032	-0.031–0.097	0.033	1060	1060	5
	*Family cohesion*	-0.106*	-0.176 –-0.032	0.036	787	787	4
	*Family conflict*	0.146*	0.075–0.217	0.036	786	786	4
	*Maternal ASPD*	0.019	-0.073–0.108	0.047	578	578	3
	*Perceived peer deviance*	0.427*	0.340–0.515	0.046	408	408	3

*Note*. Each augmented model included every component of the corresponding compact model, yet was estimated separately from the other corresponding augmented models to maximize sample size. The number of observations differ from the number of individuals when outcomes were measured at multiple follow-up time points for some individuals. Exponentiated regression weights are to be interpreted as incident rate ratios. Sex was not scaled, nor were any outcomes, excepting baseline CD, though all other predictors were standardized. Each compact model block displays parameters estimated simultaneously in the context of a single generalized linear mixed model. Each augmented model row contains parameter estimates from generalized linear mixed models regressing the outcome variable on that particular predictor after controlling for demographic factors, sample, and baseline conduct disorder. That is, each estimate was performed in the context of a separate model.

*95% credibility interval doesn’t cover zero or one for linear and exponentiated regression weights, respectively.

^†^See Table C in S1 Supplement for ethnicity contrasts.

GLMMs for repeated measures outcomes (lifetime and past year ASPD symptom counts) included random intercepts for participants. All models included random intercepts for sample. Thus, at their most complex, models were of the form:

$$f(Y_{ijt})=\alpha_{0_{ij}}+\alpha_{1}T_{t}$$

$$\alpha_{0_{ij}}=\beta_{0_{j}}+{\overset{⃑}{\beta }}_{\mathrm{cov}}^{T}{\overset{⃑}{x}}_{\mathrm{cov}}+\beta_{{\mathrm{CD}}_{j}}{\mathrm{CD}}_{ij}+\beta_{{\mathrm{PSP}}_{j}}{\mathrm{PSP}}_{ij}+U_{ij}$$

$$\beta_{0_{j}}=\gamma_{0}+V_{0_{j}}$$

$$\beta_{{\mathrm{CD}}_{j}}=\gamma_{\mathrm{CD}}+V_{{\mathrm{CD}}_{j}}$$

$$\beta_{{\mathrm{PSP}}_{j}}=\gamma_{\mathrm{PSP}}+V_{{\mathrm{PSP}}_{j}}$$

where *i* indexes individual, *j* indexes sample, *t* indexes mean deviated age(s) at follow-up(s) *T*, *cov* indicates a vector of baseline demographic covariates, *CD* indicates baseline CD symptoms, *PSP* indicates the additional psychosocial predictor measured at baseline, *Y* indicates the outcome variable, *f* indicates the link function, and roman capitals indicate random effects. The *α*, *β*, and *γ* coefficients represent regression coefficients at the level of follow-up time point, individuals, and sample, respectively. For each model, only samples that included all relevant measures were included. In addition to random intercept models, we also considered models including random effects of psychosocial predictors, but this approach failed to alter results for any outcome. For clarity, we only discuss results of random intercept models.

Additionally, we conducted sensitivity analyses to examine the potential impact of missingness of observations due to study attrition, which is known to bias parameter estimates when study dropout is non-random [62]. To this end, we constructed five replicate data sets via multivariate imputation by chain equations using predictive mean matching for continuous variables and augmented general linear models for discrete variables, using the mice package for R [63–65]. These imputed datasets are constructed under the assumption that missing data were missing-at-random (MAR) after conditioning on covariates and are heretofore referred to as the *MAR* replicates. MAR replicates were further manipulated by adding structured random offsets to each outcome to simulate different non-random dropout regimes (i.e., systematically increasing or decreasing imputed outcome measures among attrited individuals). This resulted in two additional sets of replicate datasets: *NMAR+* replicates, which simulated a positive association between attrition and outcome severity, and *NMAR-* replicates, which simulated a negative association between attrition and outcome severity. Imputed data were then analyzed using the same GLMM framework as the primary analyses and posterior distributions of parameters were averaged across replicate data sets using the *brms* R package [58]. For continuous outcomes (lifetime and past year ASPD), attrited participants’ MAR-imputed symptom counts were augmented with positive or negative binomial (*n* = 2, *p* = .5) random offsets corresponding to one average symptom differences (*SD* = .707) under the NMAR+ and NMAR- regimes, respectively. For binary outcomes (arrest after 18^th^ birthday and recent legal involvement), attrited participants’ MAR-imputed outcomes were augmented with positive or negative Bernoulli (*p* = .25) random offsets under the NMAR+ and NMAR- regimes, respectively. E.g., under the NMAR+ regime, participants who were imputed as not having experienced arrest after their 18^th^ birthday under the MAR regime then had a simulated additional 25% chance of arrest. Truncation was performed as necessary to ensure all imputed scores were plausible (e.g., negative symptom counts were not allowed). Further details and results are presented in Tables A and B in S1 Supplement, and the distributions of the observed and imputed outcomes are presented in Figures A and B in S1 Supplement.

## Results

### Descriptive statistics

The adult outcomes of the participants were severe but variable (Table 1). At follow-up, mean lifetime ASPD criteria count was 4.11 (*SD* = 1.99) and mean past year ASPD criteria count as 1.56 (*SD* = 1.54). Sixty-eight percent of participants who were under 18 years old at baseline reported being arrested after their 18^th^ birthday. Forty-eight percent of participants endorsed being on parole, on probation, or incarcerated at some point during the past five years. Attrition was moderate, with 26% of participants targeted for follow-up (310 of 1205 targeted for follow-up assessment) not participating in subsequent assessments.

### Bivariate models

Every demographic and psychosocial predictor was significantly related to at least one psychiatric or legal outcome at follow-up (Table 2). However, these estimates do not account for sample differences or other demographic factors and should be interpreted with caution.

#### Principal analyses

Effect sizes are presented as regression weights standardized with respect to the predictor but not to the outcome measure. Exceptions include sex and ethnicity contrasts, which are to be interpreted as adjusted mean differences for linear models, adjusted odds ratios for logistic models, and adjusted incident rate ratios for negative binomial models. We present 95% Bayesian credibility intervals (CI) for all coefficient estimates, which describe the 0.025 and 0.975 quantiles of their posterior distributions. An analogous notion to frequentist statistical significance at *α* = .05 can be obtained by observing whether the 95% CI covers zero or one for linear and exponentiated regression coefficients, respectively. Results for lifetime ASPD, past-year ASPD, and baseline CD are presented in Table 3; results for arrest after 18^th^ birthday, recent legal involvement, and attrition are presented in Table 4. Corresponding sensitivity analyses are presented in Tables A and B in S1 Supplement, respectively. A visual overview of how predictor effects varied across samples is presented for lifetime and past year ASPD in Figure C in S1 Supplement and for arrest and legal involvement in Figure D in S1 Supplement. Ethnicity contrasts for all outcomes are presented in Table C in S1 Supplement.

**Table 4:** Generalized linear mixed model results: Legal outcomes and attrition.

**Arrest after 18**^**th**^ **birthday**		**e**^*β*^	**95% CI**	***SE***	Observations	Individuals	Samples
Compact model							
	*Baseline CD*	1.193	0.981–1.450	0.100	743	743	5
	*Age baseline*	1.020	0.783–1.315	0.130	743	743	5
	*Age follow-up*	1.081	0.877–1.337	0.107	743	743	5
	*Sex (male vs*. *female)*	3.546*	2.303–5.506	0.220	743	743	5
	*Ethnicity*^†^	—	—	—	743	743	5
Augmented models							
	*Substance vulnerability*	0.938	0.768–1.145	0.102	743	743	5
	*Block design*	0.828	0.671–1.029	0.111	660	660	5
	*Vocabulary*	0.742*	0.595–0.919	0.112	660	660	5
	*Family cohesion*	0.979	0.792–1.209	0.109	509	509	4
	*Family conflict*	0.955	0.766–1.194	0.112	508	508	4
	*Maternal ASPD*	1.084	0.751–1.582	0.191	258	258	3
	*Perceived peer deviance*	0.790	0.451–1.377	0.281	247	247	3
**Recent legal involvement**		**e**^*β*^	**95% CI**	***SE***	Observations	Individuals	Samples
Compact model							
	*Baseline CD*	1.099	0.921–1.311	0.090	741	741	5
	*Age baseline*	0.910	0.741–1.116	0.106	741	741	5
	*Age follow-up*	0.839	0.662–1.064	0.120	741	741	5
	*Sex (male vs*. *female)*	3.083*	2.042–4.693	0.212	741	741	5
	*Ethnicity*^†^	—	—	—	741	741	5
Augmented models							
	*Substance vulnerability*	1.126	0.933–1.351	1.126	741	741	5
	*Block design*	0.832	0.686–1.008	0.832	690	690	5
	*Vocabulary*	0.835	0.657–1.025	0.835	690	690	5
	*Family cohesion*	1.078	0.894–1.298	1.078	553	553	4
	*Family conflict*	0.942	0.774–1.142	0.942	552	552	4
	*Maternal ASPD*	1.215	0.880–1.673	1.215	229	229	3
	*Perceived peer deviance*	1.152	0.768–1.728	1.152	189	189	3
**Attrition**		**e**^*β*^	**95% CI**	***SE***	Observations	Individuals	Samples
Compact model							
	*Baseline CD*	1.107	0.971–1.264	0.068	1205	1205	5
	*Age baseline*	0.858	0.735–1.000	0.079	1205	1205	5
	*Sex (male vs*. *female)*	1.157	0.797–1.687	0.191	1205	1205	5
	*Ethnicity*^†^	—	—	—	1205	1205	5
Augmented models							
	*Substance vulnerability*	0.926	0.800–1.073	0.075	1205	1205	5
	*Block design*	0.952	0.810–1.108	0.080	1059	1059	5
	*Vocabulary*	0.784*	0.666–0.922	0.083	1060	1060	5
	*Family cohesion*	0.831	0.691–1.009	0.096	786	786	4
	*Family conflict*	1.103	0.912–1.342	0.099	787	787	4
	*Maternal ASPD*	1.148	0.906–1.465	0.121	461	461	3
	*Perceived peer deviance*	0.883	0.648–1.209	0.159	408	408	3

*Note*. Each augmented model included every component of the corresponding compact model, yet was estimated separately from the other corresponding augmented models to maximize sample size. Each augmented model row contains parameter estimates from generalized linear mixed models regressing the outcome variable on that particular predictor after controlling for demographic factors, sample, and baseline conduct disorder. That is, each estimate was performed in the context of a separate model. See caption of Table 3 for further details.

*95% credibility interval doesn’t cover one.

^†^See Table C in S1 Supplement for ethnicity contrasts

#### Lifetime ASPD

In the compact model, sex and baseline CD explained independent outcome variance such that adjusted mean symptom counts were 0.919 greater in males (95% CI: 0.609–1.221) and 0.462 per unit standard deviation increase in baseline CD symptom (95% CI: 0.342–0.585). Additionally, average symptom count was lowest among non-Latino Caucasian participants, who displayed between 0.089 and 1.094 fewer symptoms than non-Latino African-American participants (Table D in S1 Supplement). In the augmented models, no psychosocial predictors explained unique outcome variance after accounting for baseline demographics, baseline CD, and age at follow-up (Table 3). Sensitivity analyses suggested that estimates for the effects of sex and baseline CD were robust across multiple potential patterns of differential attrition (Table A in S1 Supplement).

#### Past year ASPD

In the compact model, sex and baseline CD again explained independent outcome variance (*e*^*β*^ = 1.264, 95% CI: 1.091–1.462; *e*^*β*^ = 1.141, 95% CI: 1.066–1.190; respectively). Additionally, marginal incidence rate of symptom counts was greater among African-American participants as compared to other groups (Table D in S1 Supplement). Again, no psychosocial predictors explained unique outcome variance after accounting for baseline demographics, baseline CD, and age at follow-up (Table 3). Sensitivity analyses again suggested that estimates for the effects of sex and baseline CD were robust across multiple potential patterns of differential attrition (Table A in S1 Supplement). Further, MAR sensitivity analyses, which assumed attrition and past year ASPD were conditionally independent given the covariates, suggested possible independent contributions of family conflict and family cohesion (*e*^*β*^ = 1.071, 95% CI: 1.017–1.127; *e*^*β*^ = 0.947, 95% CI: 0.868–1.009; respectively; Table A in S1 Supplement).

#### Arrest after 18^th^ birthday

In the compact model, sex (but not CD) explained independent outcome variance (*e*^*β*^ = 3.546, 95% CI: 2.303–5.506). Conditional marginal odds of arrest did not differ across race/ethnicity categories in the context of the compact model (Table C in S1 Supplement). Among additional predictors, vocabulary and bock design evidenced independent (negative) contributions to adjusted odds of arrest in the context of the augmented models (*e*^*β*^ = 0.742, 95% CI: 0.595–0.919; Table 4). Sensitivity analyses supported the contributions of sex, block design, and vocabulary across multiple non-random attrition regimes (Table B in S1 Supplement).

#### Recent legal involvement

Consistent with the results for arrest, only sex evidenced partial associations in the compact model (*e*^*β*^ = 3.083, 95% CI: 2.042–4.693). Conditional marginal odds of recent legal involvement were substantially elevated in non-Latino African-American and non-Caucasian Latino participants relative to non-Latino Caucasian participants in the context of the compact model (Table C in S1 Supplement). Additionally, both block design and vocabulary evidenced trending associations with decreased odds of recent legal involvement (*e*^*β*^ = 0.832, 95% CI: 0.686–1.008; *e*^*β*^ = 0.835, 95% CI: 0.657–1.025; respectively; Table 4), with sensitivity analyses again supporting the contributions of sex, block design, and vocabulary across multiple non-random attrition regimes and additionally suggesting that adjusted odds decreased with age at follow-up assessment (Table B in S1 Supplement).

#### Attrition

Among all psychosocial predictors, only vocabulary evidenced independent contributions to outcome variance such standard deviation increases in vocabulary score decreased odds of attrition by a factor of 0.784 (95% CI: 0.666–0.922), though a similar trend was present for family cohesion (*e*^*β*^ = 0.831, 95% CI: 0.691–1.009; Table 4). Ethnicity contrasts suggested that non-Latino African-American had greater marginal odds of attriting compared to all other racial/ethnic categories (Table C in S1 Supplement).

### Post-hoc analyses

#### Cross-sectional associations with baseline CD

To further understand the differing patterns of results between zero-order models (where most psychosocial predictors exhibited associations with adult outcomes; Table 2) and multivariate models (where relatively few psychosocial predictors explained outcome variation after controlling for baseline CD and demographic factors; Tables 3 and 4), we examined the extent to which individual psychosocial predictors explained concurrent CD symptoms after controlling for age, sex, and ethnicity. The effect of each psychosocial predictor was examined in the context of a linear mixed effects regression including random intercepts across samples.

Male participants’ marginal symptom counts were between 0.222 and 0.512 standard deviations greater than female participants’ (Table 3), though no differences were evident across racial/ethnic categories (Table C in S1 Supplement). Augmented model results (Table 3) were wholly consistent with unconditional bivariate analyses (Table 2). That is, accounting for sample, age, sex, and ethnicity did not alter evidence for contributions of substance vulnerability, family cohesion, family conflict, and perceived peer deviance, whereas block design, vocabulary, and maternal ASPD did not evidence contributions to baseline CD in either context (Tables 2 and 3).

#### Contributions of aggressive and non-aggressive CD symptom clusters

Given the substantial interest in aggressive versus non-aggressive CD symptom clusters [20,22,23,66], as well as the lack of evidence for independent contributions of baseline CD to arrest after 18^th^ birthday or legal in the context of multivariate models (Table 3) despite evidencing zero-order bivariate associations (Table 2), we examined the independent contributions of symptom clusters to each longitudinal outcome. Specifically, for each outcome, we simultaneously estimated contributions of aggressive and non-aggressive symptoms while accounting for age, sex, ethnicity, and sample. Both aggressive and non-aggressive CD symptoms evidenced independent contributions to lifetime ASPD whereas only aggressive CD symptoms evidenced independent contributions to past year ASPD (Table D in S1 Supplement). Further, whereas slopes for aggressive versus non-aggressive symptom clusters did not differ for lifetime ASPD (95% CI: -0.190–0.314), differences for past year ASPD were less certain (95% CI: -0.006–0.213). No independent contributions of either symptom cluster were apparent for arrest or legal involvement and follow-up analyses did not provide evidence for an interaction between symptom clusters across all outcomes (Table D in S1 Supplement).

#### Partial effects of intelligence measures

Both principal analyses (Table 4) and sensitivity analyses (Table B in S1 Supplement) of arrest and recent legal involvement suggested possible independent contributions of design and vocabulary measures evidenced negative partial associations with current ASPD symptom count, arrest after 18^th^ birthday, and recent legal involvement beyond baseline CD and demographic factors (Tables 3 and 4). Because these two measures are correlated (Table 2), we reran the relevant principal analyses, simultaneously modeling the partial effects of both intelligence measures beyond demographic variables and baseline CD. For both arrest after 18^th^ birthday and recent legal involvement, vocabulary, but not block design evidenced independent contributions beyond one another, sex, age, ethnicity, and sample (Table D in S1 Supplement). However, 95% credibility intervals for slope differences covered zero in both cases (95% CI: -0.467–0.209, 95% CI: -0.507–0.162, for arrest and legal involvement, respectively). Follow-up analyses did not provide evidence for an interaction between measures across all outcomes (Table D in S1 Supplement).

#### Vocabulary-by-maternal ASPD interaction

Further analyses were motivated by the lack of evidence for contributions of maternal ASPD across all outcomes (Tables 3 and 4), evidence for independent contributions of vocabulary to odds of arrest after 18^th^ birthday and recent legal involvement, and previous research suggesting that the association between verbal deficits and future EP is moderated by parental ASPD [18]. We thus estimated additional models including terms for maternal ASPD, vocabulary score, and their product, as well as baseline CD symptoms and demographic factors. We found no evidence of such an interaction across any of the adult outcomes (Table D in S1 Supplement).

## Discussion

The present study examined the degree to which multiple available theoretically-motivated predictors explained unique variance in concurrent and adult outcomes in a longitudinal, multi-site study of adolescents ascertained for severe EP. Our results pertain to the practical problem of selecting additional risk factors to measure on top of information provided by demographic variables and baseline CD to assess risk for persistence of EP into adulthood in intervention and prevention settings. We discuss the contributions of demographic predictors, baseline CD, and psychosocial predictors in turn.

Despite the initial severity of the samples, adult outcomes were variable—e.g., 51.94% of individuals reported recent legal involvement at follow-up assessment. Prominent sex differences were apparent across all outcomes other than attrition. For example, after controlling for age at baseline, age(s) at follow-up(s), ethnicity, and baseline CD, males’ odds of arrest after 18^th^ birthday and incident rates of past-year ASPD symptoms were 3.546 and 1.263 times greater than those of females, respectively (Tables 3 and 4). Non-Latino African-American participants were broadly at greater risk for negative outcomes, after accounting for baseline CD and other predictors, though these effects varied substantially across outcomes (Table C in S1 Supplement). Reasons for these differences are unclear, but are likely to reflect systematic variation in unmeasured socioeconomic factors. Moreover, non-Latino African-American participants demonstrated elevated rates of attrition, which sensitivity analyses suggest might have influenced results (Tables A and B in S1 Supplement), complicating interpretations of effects of ethnicity on psychiatric and legal outcomes. Age at baseline assessment failed to consistently predict any outcomes variables, which is unsurprising as it is a poor proxy for age-of-onset of EP.

Baseline CD independently predicted both lifetime and past-year ASPD criteria count, which is consistent with the conceptualization of CD as a developmental precursor to ASPD. Independent associations between CD and negative legal outcomes were directionally consistent with ASPD results but subject to greater uncertainty (Tables 4, Table B in S1 Supplement). Follow-up analyses suggested that contributions of aggressive and non-aggressive symptoms to lifetime ASPD were indistinguishable, whereas only aggressive symptoms appeared to contribute to past-year ASPD. These results are at odds with a recent study reporting that only aggressive CD symptoms contributed to lifetime ASPD in a sample of 254 boys recruited from an economically distressed area in Pennsylvania [21]. However, lifetime ASPD symptoms might be a poor indicator of persistence that simply reflected the severity of baseline CD among participants in the present study. Our results for past year ASPD are indeed congruent with greater contributions of aggressive symptoms to persistence of EP.

Despite significant zero-order associations between many psychosocial predictors and outcome variables (Table 2), as well as evidence for independent associations between these predictors and baseline CD (Table 3), few were unique predictors after accounting for demographic characteristics and baseline CD (Tables 3 and 4). Moreover, some variables associated with concurrent CD in adolescence (i.e., substance abuse and perceived peer deviance), failed to explain independent variance in any adult outcomes beyond that explained by baseline CD and demographics. On the other hand, vocabulary and block design, which both had significantly variable cross-sectional associations with CD in adolescence across samples, were the only predictors that predicted adult legal outcomes with moderate consistency. For other predictors, patterns were less clear; e.g., family conflict contributed independent variance to baseline CD and past year ASPD, but not to lifetime ASPD, with sensitivity analyses suggesting that study attrition may have attenuated coefficient estimates.

Follow-up analyses examining the simultaneous partial effects of intelligence measures suggested that, after accounting for the outcome variance explained by variation common to vocabulary and block design, deficits in vocabulary still predicted negative adult legal outcomes. One possible explanation for this divergence is that vocabulary deficits might impair individuals’ ability to effectively navigate the legal system, though we are hesitant to make such claims, given the exploratory nature of these analyses and lack of a comprehensive measure of socioeconomic status (SES) (Table 3). Further, examining the posterior distribution of vocabulary/performance slope differences did not suggest robust differences in effects.

There are several limitations in the present investigation. As mentioned above, study attrition was moderate (26%; Table 1), differed substantially between ethnic groups (Table C in S1 Supplement), and possibly biased estimates effects of family cohesion and family conflict (Table A in S1 Supplement). Second, as outcomes were measured during at most two follow-up assessments per individual, a latent growth modeling approach, which would have allowed us to differentiate between factors affecting mean levels versus changes in ASPD severity over time, was not possible. Third, not all measures were administered for every sample (Table 1), diminishing effective sample size (and hence statistical power) for a number of covariates. Fourth, despite considerable interest in the contributions in possible connections between callous-unemotional (CU) traits and EP [21,67,68], such measures were unavailable for the present sample, though the utility of CU traits in examining *persistence* of EP is a point of controversy [67,69,70]. Fifth, our measures of legal outcomes did not distinguish between qualitatively different types of crime (e.g., violent versus non-violent crimes), perhaps masking type-specific contributions of psychosocial predictors. For example, previous work suggests that SUD among detained adolescents increases risk for substance-related recidivism [71]; it is therefore possible that failure to distinguish between substance- and non-substance-related legal outcomes might partially underlie the failure to detect independent contributions of substance vulnerability in the present investigation. Sixth, and perhaps most crucially, a comprehensive measure of SES was unavailable, which was unfortunate given the known associations between SES, ethnicity, and intelligence measures. Finally, we acknowledge that collapsing across diverse samples may have reduced are ability to detect sample-specific effects of individual predictors, even after adjusting for sample differences. However, allowing slopes associated with psychosocial variables to vary by sample failed to substantially alter our conclusions (see Figures C-D in S1 Supplement for a graphic presentation). In the search for robust predictors of unique outcome variance, we have focused on variables with the potential to be valuable across a wide of prevention and intervention efforts and believe this approach to be practical to that end.

The present study examined the independent predictive contributions of a wide array of candidate risk factors for the persistence of adolescent EP into adulthood. Although we considered a large variety of theoretically-derived psychosocial predictors, we were ultimately limited to those available in the present sample and thus cannot claim to have covered the breadth of candidate risk factors suggested in the literature. Specifically, in addition to previously discussed limitations, we lacked comprehensive measures of personality traits, psychiatric comorbidity, psychopathy, emotional and behavioral regulation, parenting practices, and community level risk factors (e.g., crime rates), each of which has been suggested to increase risk for persistent EP [3,71–73]. Still, given the spectrum of predictors considered, our results inform the development of prevention, intervention, and public health initiatives, as we examined a sample of youth with severe externalizing problems who were already “naturally” ascertained by clinical or correctional programs. Individuals such as our participants, virtue of having already attracted the attention of public institutions (be they clinical or correctional), are clear targets for public health initiatives.

Developmental taxonomic theory, the dominant theoretical perspective in the longitudinal study of EP, predicts that the continuity of these behaviors into adulthood should reflect psychosocial and socioeconomic factors. Many of these psychosocial variables were associated with unadjusted legal outcomes in adulthood (Table 2) and explained unique variation in CD severity during adolescence (Table 3). However, parsimony favored models only including demographic variables and, in the case of ASPD, baseline CD. Intelligence measures were the only additional variables to consistently explain independent variation across legal outcomes, but it is unclear how these results would change in the presence of an adequate measure of SES. We speculate that unmeasured socioeconomic factors may underlie much of the observed heterogeneity in persistence of EP, but our data did not permit further investigation of this hypothesis.

We suggest targets for future research efforts based on our findings. Given that the observed negative associations between vocabulary and legal outcomes may reflect underlying socioeconomic disparities, these associations should be examined after accounting for potential socioeconomic confounds related to educational attainment. Alternatively, a discordant-sibling approach would address this question without fine-grained measurements of socioeconomic factors. Additionally, our evidence is congruent with the taxonomic theory’s notion that the stability of EP is linked to the “extremity” of EP [3]. That is, greater severity of EP as indexed by CD symptoms was associated with negative adult outcomes above all other factors. On the other hand, our results suggest that examining underlying clusters of psychosocial and neurocognitive risk factors in adolescence beyond CD severity and demographic factors may not be useful in predicting adult outcomes. To our understanding, current empirical results remain agnostic with regard to whether persistence versus desistence of EP in adulthood reflects unique clusters of risk factors or relates monotonically to EP severity in adolescence. Particularly, the latent class modeling analyses underlying the majority of trajectory based work are fundamentally exploratory, and a posteriori comparisons of groups of individuals differentiated by these methods are difficult to interpret [7,8]. Future investigations should directly compare confirmatory discrete (i.e., class-based) and continuous (i.e., severity-based) models of persistent EP. Finally, given the increasing availability of large datasets and our growing abilities to link diverse data sources (e.g., court records and electronic health records), prediction-focused research (in contrast to explanation-focused research) is growing in feasibility [74]. Though these approaches are not mutually exclusive, we suggest that a future emphasis on maximizing out-of-sample predictive performance with respect to persistence of EP has the potential to help determine what additional information will be most useful to clinical and legal practitioners working with at-risk adolescents.

## Supporting information

S1 Supplement Supplementary Tables A-D and supplementary Figures A-D.
(DOCX)
