# Supplementary material for: Predictors of adult outcomes in clinically- and legally-ascertained youth with externalizing problems
Source: PLoS One. 2018 Nov 1;13(11):e0206442. doi: 10.1371/journal.pone.0206442 (PMC6211688; doi:10.1371/journal.pone.0206442)
Supplement: S1 Supplement — Supplementary Tables A-D and supplementary Figures A-D. (DOCX) [file pone.0206442.s001.docx]

Contents

[Table A. Sensitivity to non-random missingness: externalizing outcomes 2](#_Toc514529335)

[Table B. Sensitivity to non-random missingness: legal outcomes 3](#_Toc514529336)

[Table C. Race/ethnicity contrasts 4](#_Toc514529337)

[Table D. Exploratory analyses 5](#_Toc514529338)

[Figure A. Distributions of observed and imputed outcomes: past year and lifetime ASPD symptoms 6](#_Toc514529339)

[Figure B. Distributions of observed and imputed outcomes: arrest after 18^th^ birthday and recent legal involvement 7](#_Toc514529340)

[Figure C. Predictor slopes by sample: past year and lifetime ASPD symptoms 8](#_Toc514529341)

[Figure D. Predictor slopes by sample: arrest after 18th birthday and recent legal involvement 9](#_Toc514529342)

Table A. Sensitivity to non-random missingness: externalizing outcomes

|  | | **MAR**  ***Observations lost to attrition missing at random*** | | |  | **MNAR+**  ***Positive association between severity and attrition*** | | | | **MNAR-**  ***Negative association between severity and attrition*** | | | |
| --- | --- | --- | --- | --- | --- | --- | --- | --- | --- | --- | --- | --- | --- |
| **Lifetime ASPD** | | ***β*** | **95% CI** | ***SE*** |  | | ***β*** | **95% CI** |  | | ***β*** | **95% CI** |  |
| Compact model | *Baseline CD* | 0.479^*^ | 0.366 – 0.587 | 0.057 |  | | 0.471^*^ | 0.348 – 0.593 |  | | 0.387^*^ | 0.268 – 0.505 |  |
|  | *Age baseline* | 0.026 | -0.063 – 0.113 | 0.045 |  | | -0.066 | -0.170 – 0.039 |  | | 0.032 | -0.072 – 0.137 |  |
|  | *Age follow-up* | -0.018 | -0.064 – 0.023 | 0.024 |  | | 0.076^*^ | 0.036 – 0.127 |  | | -0.027 | -0.064 – 0.012 |  |
|  | *Sex (male vs. female)* | 0.800^*^ | 0.453 – 1.121 | 0.178 |  | | 0.877^*^ | 0.570 – 1.179 |  | | 0.788^*^ | 0.476 – 1.096 |  |
|  | *Ethnicity* | — | — | — |  | | — | — |  | | — | — |  |
| Augmented models | *Substance vulnerability* | 0.013 | -0.170 – 0.202 | 0.109 |  | | 0.035 | -0.101 – 0.165 |  | | 0.011 | -0.111 – 0.132 |  |
|  | *Block design* | 0.012 | -0.112 – 0.137 | 0.065 |  | | 0.036 | -0.103 – 0.174 |  | | 0.032 | -0.098 – 0.164 |  |
|  | *Vocabulary* | -0.051 | -0.259 – 0.104 | 0.100 |  | | -0.023 | -0.159 – 0.113 |  | | -0.010 | -0.147 – 0.128 |  |
|  | *Family cohesion* | -0.088 | -0.216 – 0.066 | 0.072 |  | | -0.049 | -0.188 – 0.095 |  | | -0.066 | -0.206 – 0.074 |  |
|  | *Family conflict* | 0.073 | -0.080 – 0.203 | 0.073 |  | | 0.112 | -0.029 – 0.255 |  | | 0.110 | -0.033 – 0.248 |  |
|  | *Maternal ASPD* | 0.057 | -0.126 – 0.303 | 0.113 |  | | 0.025 | -0.162 – 0.213 |  | | 0.039 | -0.148 – 0.233 |  |
|  | *Perceived peer deviance* | 0.179 | -0.004 – 0.360 | 0.094 |  | | 0.187 | -0.027 – 0.400 |  | | 0.178 | -0.038 – 0.391 |  |
| **Past year ASPD** | | **e***^β^* | **95% CI** | ***SE*** |  | | **e***^β^* | **95% CI** |  | | **e***^β^* | **95% CI** |  |
| Compact model | *Baseline CD* | 1.136^*^ | 1.078 – 1.201 | 0.028 |  | | 1.124^*^ | 1.068 – 1.183 |  | | 1.119^*^ | 1.059 – 1.183 |  |
|  | *Age baseline* | 1.036 | 0.988 – 1.084 | 0.024 |  | | 0.976 | 0.930 – 1.023 |  | | 1.033 | 0.985 – 1.084 |  |
|  | *Age follow-up* | 0.971 | 0.954 – 0.987 | 0.009 |  | | 1.021 | 1.005 – 1.038 |  | | 0.963^*^ | 0.945 – 0.983 |  |
|  | *Sex (male vs. female)* | 1.225^*^ | 1.010 – 1.433 | 0.092 |  | | 1.199^*^ | 1.032 – 1.382 |  | | 1.215^*^ | 1.042 – 1.409 |  |
|  | *Ethnicity* | — | — | — |  | | — | — |  | | — | — |  |
| Augmented models | *Substance vulnerability* | 1.001 | 0.953 – 1.051 | 0.025 |  | | 0.992 | 0.941 – 1.045 |  | | 0.993 | 0.939 – 1.049 |  |
|  | *Block design* | 1.054 | 0.991 – 1.128 | 0.035 |  | | 1.056 | 0.993 – 1.122 |  | | 1.059 | 0.998 – 1.125 |  |
|  | *Vocabulary* | 1.020 | 0.964 – 1.075 | 0.028 |  | | 1.005 | 0.948 – 1.065 |  | | 1.005 | 0.941 – 1.074 |  |
|  | *Family cohesion* | 0.947 | 0.868 – 1.009 | 0.039 |  | | 0.964 | 0.908 – 1.023 |  | | 0.961 | 0.903 – 1.024 |  |
|  | *Family conflict* | 1.071^*^ | 1.017 – 1.127 | 0.026 |  | | 1.056 | 0.990 – 1.126 |  | | 1.053 | 0.987 – 1.124 |  |
|  | *Maternal ASPD* | 1.043 | 0.925 – 1.202 | 0.078 |  | | 1.009 | 0.923 – 1.103 |  | | 1.015 | 0.928 – 1.108 |  |
|  | *Perceived peer deviance* | 0.964 | 0.890 – 1.047 | 0.042 |  | | 0.964 | 0.873 – 1.063 |  | | 0.963 | 0.869 – 1.065 |  |

*Note.* Models are structurally the same as those presented in Table 3, though each analysis was performed five times on five replicate imputed data sets; coefficients above reflect pooled posterior distributions across replicates. Replicate datasets were constructed via multivariate imputation by chain equations using predictive mean matching for continuous variables and augmented general linear models for discrete variables. *MAR* analyses were performed on these data, and are valid under the assumption that attrition and outcome severity are conditionally independent given the covariates. *MAR* replicates were further manipulated by adding structured random offsets to each outcome to simulate different non-random attrition regimes. *NMAR+* replicates result from simulating a positive association between attrition and outcome severity, and *NMAR-* replicates result from simulating a negative association between attrition and outcome severity. This was accomplished by augmenting attrited participants’ *MAR*-imputed symptom counts with positive or negative binomial (n = 2, p = .5) random offsets corresponding to one average symptom differences (SD = .707) under the *NMAR+* and *NMAR-* regimes, respectively (See Figure S1 for distributions of observed data and imputed data under each regime).

^*^ 95% credibility interval doesn’t cover zero or one for linear and exponentiated regression weights, respectively.

Table B. Sensitivity to non-random missingness: legal outcomes

|  | | **MAR**  ***Observations lost to attrition missing at random*** | | | |  | **MNAR+**  ***Positive association between severity and attrition*** | | | | **MNAR-**  ***Negative association between severity and attrition*** | | | | |
| --- | --- | --- | --- | --- | --- | --- | --- | --- | --- | --- | --- | --- | --- | --- | --- |
| **Arrest after 18^th^ birthday** | | ***e^β^*** | **95% CI** | ***SE*** |  | | | ***e^β^*** | **95% CI** |  | | ***e^β^*** | **95% CI** |  |  |
| Compact model | *Baseline CD* | 1.157 | 0.963 – 1.404 | 0.096 |  | | | 1.144 | 0.966 – 1.354 |  | | 1.100 | 0.953 – 1.270 |  |  |
|  | *Age baseline* | 0.986 | 0.762 – 1.258 | 0.130 |  | | | 1.035 | 0.844 – 1.269 |  | | 1.122 | 0.937 – 1.349 |  |  |
|  | *Age follow-up* | 1.080 | 0.888 – 1.315 | 0.101 |  | | | 1.150 | 0.940 – 1.405 |  | | 0.849 | 0.709 – 1.019 |  |  |
|  | *Sex (male vs. female)* | 3.516^*^ | 2.405 – 5.081 | 0.192 |  | | | 3.335^*^ | 2.331 – 4.823 |  | | 2.877^*^ | 2.028 – 4.130 |  |  |
|  | *Ethnicity* | — | — | — |  | | | — | — |  | | — | — |  |  |
| Augmented models | *Substance vulnerability* | 0.942 | 0.787 – 1.126 | 0.092 |  | | | 1.014 | 0.864 – 1.194 |  | | 0.988 | 0.838 – 1.173 |  |  |
|  | *Block design* | 0.813^*^ | 0.678 – 0.972 | 0.093 |  | | | 0.813^*^ | 0.676 – 0.973 |  | | 0.879 | 0.748 – 1.052 |  |  |
|  | *Vocabulary* | 0.771^*^ | 0.632 – 0.967 | 0.109 |  | | | 0.786^*^ | 0.648 – 0.971 |  | | 0.865^*^ | 0.702 – 1.088 |  |  |
|  | *Family cohesion* | 1.006 | 0.849 – 1.199 | 0.088 |  | | | 1.041 | 0.889 – 1.220 |  | | 1.095 | 0.928 – 1.301 |  |  |
|  | *Family conflict* | 0.974 | 0.818 – 1.154 | 0.087 |  | | | 0.963 | 0.824 – 1.127 |  | | 0.949 | 0.800 – 1.133 |  |  |
|  | *Maternal ASPD* | 1.134 | 0.885 – 1.453 | 0.126 |  | | | 1.167 | 0.890 – 1.556 |  | | 1.092 | 0.897 – 1.335 |  |  |
|  | *Perceived peer deviance* | 1.051 | 0.731 – 1.577 | 0.190 |  | | | 1.046 | 0.708 – 1.685 |  | | 1.072 | 0.728 – 1.535 |  |  |
| **Recent legal involvement** | | ***e^β^*** | **95% CI** | ***SE*** |  | | | ***e^β^*** | **95% CI** |  | | ***e^β^*** | **95% CI** |  |  |
| Compact model | *Baseline CD* | 1.125 | 0.968 – 1.323 | 0.08 |  | | | 1.119 | 0.966 – 1.298 |  | | 1.091 | 0.954 – 1.250 |  |  |
|  | *Age baseline* | 0.862 | 0.705 – 1.050 | 0.102 |  | | | 0.842 | 0.703 – 1.001 |  | | 0.924 | 0.763 – 1.097 |  |  |
|  | *Age follow-up* | 0.831^*^ | 0.703 – 0.977 | 0.084 |  | | | 0.916 | 0.751 – 1.111 |  | | 0.774^*^ | 0.636 – 0.927 |  |  |
|  | *Sex (male vs. female)* | 2.765^*^ | 1.940 – 3.973 | 0.183 |  | | | 2.650^*^ | 1.837 – 3.824 |  | | 2.576^*^ | 1.783 – 3.810 |  |  |
|  | *Ethnicity* | — | — | — |  | | | — | — |  | | — | — |  |  |
| Augmented models | *Substance vulnerability* | 1.150 | 0.978 – 1.370 | 0.088 |  | | | 1.130 | 0.962 – 1.325 |  | | 1.160 | 0.980 – 1.376 |  |  |
|  | *Block design* | 0.854^*^ | 0.738 – 0.984 | 0.073 |  | | | 0.839^*^ | 0.722 – 0.974 |  | | 0.919 | 0.800 – 1.050 |  |  |
|  | *Vocabulary* | 0.787^*^ | 0.660 – 0.953 | 0.096 |  | | | 0.779^*^ | 0.657 – 0.928 |  | | 0.834^*^ | 0.711 – 0.977 |  |  |
|  | *Family cohesion* | 1.062 | 0.885 – 1.256 | 0.089 |  | | | 1.030 | 0.822 – 1.236 |  | | 1.090 | 0.898 – 1.290 |  |  |
|  | *Family conflict* | 0.939 | 0.755 – 1.136 | 0.107 |  | | | 0.944 | 0.792 – 1.122 |  | | 0.924 | 0.763 – 1.111 |  |  |
|  | *Maternal ASPD* | 1.143 | 0.892 – 1.465 | 0.131 |  | | | 1.118 | 0.890 – 1.414 |  | | 1.120 | 0.917 – 1.364 |  |  |
|  | *Perceived peer deviance* | 1.287 | 0.947 – 1.718 | 0.154 |  | | | 1.266 | 0.928 – 1.701 |  | | 1.265 | 0.915 – 1.711 |  |  |

*Note.* Models are structurally the same as those presented in Table 4, though each analysis was performed five times on five replicate imputed data sets; coefficients above reflect pooled posterior distributions across replicates. Replicate datasets were constructed via multivariate imputation by chain equations using predictive mean matching for continuous variables and augmented general linear models for discrete variables. *MAR* analyses were performed on these data, and are valid under the assumption that attrition and outcome severity are conditionally independent given the covariates. *MAR* replicates were further manipulated by adding structured random offsets to each outcome to simulate different non-random attrition regimes. *NMAR+* replicates result from simulating a positive association between attrition and outcome severity, and *NMAR-* replicates result from simulating a negative association between attrition and outcome severity. This was accomplished by augmenting attrited participants’ *MAR*-imputed symptom counts with positive or negative Bernoulli (p = .25) random offsets (See Figure S2 for distributions of observed data and imputed data under each regime). Attrited participants who were imputed as not having experienced arrest under the *MAR* regime had a 25% chance being imputed as arrested under the *NMAR+* regime. Likewise, attrited participants who were imputed as having experienced recent legal involvement under the *MAR* regime had a 25% chance being imputed as not experiencing recent legal involvement under the *NMAR-* regime.

^*^ 95% credibility interval doesn’t cover zero or one for linear and exponentiated regression weights, respectively.

Table C. Race/ethnicity contrasts

| **Lifetime ASPD** | *African-American (non-Latino)* | *Latino (non-Caucasian)* | *Caucasian (non-Latino)* |
| --- | --- | --- | --- |
| *Latino* | 0.520 [-0.017 – 1.040] |  |  |
| *Caucasian (non-Latino)* | 0.585 [0.089 – 1.094]^*^ | 0.070 [-0.215 – 0.356] |  |
| *Multiple/Other/Unknown* | 0.241 [-0.330 – 0.801] | -0.279 [-0.666 – 0.110] | -0.353 [-0.709 – 0.003] |
|  |  |  |  |
| **Past year ASPD** | *African-American (non-Latino)* | *Latino (non-Caucasian)* | *Caucasian (non-Latino)* |
| *Latino* | 1.305 [1.051 – 1.620]^*^ |  |  |
| *Caucasian (non-Latino)* | 1.372 [1.110 – 1.697]^*^ | 1.051 [0.920 – 1.193] |  |
| *Multiple/Other/Unknown* | 1.365 [1.068 – 1.740]^*^ | 1.046 [0.869 – 1.261] | 0.994 [0.839 – 1.176] |
|  |  |  |  |
| **Baseline CD** | *African-American (non-Latino)* | *Latino (non-Caucasian)* | *Caucasian (non-Latino)* |
| *Latino* | -0.242 [-0.459 – -0.020] ^*^ |  |  |
| *Caucasian (non-Latino)* | -0.154 [-0.359 – 0.055] | 0.089 [-0.039 – 0.227] |  |
| *Multiple/Other/Unknown* | -0.257 [-0.500 – -0.011] ^*^ | -0.017 [-0.204 – 0.174] | -0.104 [-0.283 – 0.069] |
|  |  |  |  |
| **Arrest after 18^th^ birthday** | *African-American (non-Latino)* | *Latino (non-Caucasian)* | *Caucasian (non-Latino)* |
| *Latino* | 0.683 [0.309 – 1.532] |  |  |
| *Caucasian (non-Latino)* | 1.074 [0.497 – 2.246] | 1.590 [0.985 – 2.549] |  |
| *Multiple/Other/Unknown* | 0.950 [0.397 – 2.291] | 1.402 [0.736 – 2.614] | 0.885 [0.510 – 1.529] |
|  |  |  |  |
| **Recent legal involvement** | *African-American (non-Latino)* | *Latino (non-Caucasian)* | *Caucasian (non-Latino)* |
| *Latino* | 1.628 [0.766 – 3.400] |  |  |
| *Caucasian (non-Latino)* | 2.586 [1.264 – 5.507]^*^ | 1.568 [1.042 – 2.433]^*^ |  |
| *Multiple/Other/Unknown* | 2.575 [1.131 – 5.803]^*^ | 1.589 [0.886 – 2.803] | 1.012 [0.609 – 1.674] |
|  |  |  |  |
| **Attrition** | *African-American (non-Latino)* | *Latino (non-Caucasian)* | *Caucasian (non-Latino)* |
| *Latino* | 1.548 [0.926 – 2.560]^*^ |  |  |
| *Caucasian (non-Latino)* | 3.086 [1.898 – 4.999]^*^ | 2.017 [1.447 – 2.826] |  |
| *Multiple/Other/Unknown* | 4.230 [2.314 – 7.925]^*^ | 2.758 [1.662 – 4.699] | 1.366 [0.850 – 2.256] |

*Note*: Ethnicity contrasts corresponding to the compact models presented in Tables 3-4. Rows indicate the referent group and columns indicate the target group. E.g., the first cell of the first row of the lifetime ASPD contrasts reflects the posterior distribution of adjusted lifetime ASPD symptom count between non-Latino African American participants and Latino participants. Past year ASPD contrasts reflect symptom incident rate ratios. Baseline CD contrasts are standardized. Arrest, legal involvement, and attrition contrasts are adjusted odds ratios.

Table D. Exploratory analyses: symptom clusters, intelligence measures, interactions.

| **Lifetime ASPD** | | ***β*** | **95% CI** | ***SE*** |  | **Past year ASPD** | | ***e^β^*** | **95% CI** | ***SE*** |
| --- | --- | --- | --- | --- | --- | --- | --- | --- | --- | --- |
|  | *Aggressive CD symptoms* | 0.296^*^ | 0.158 – 0.432 | 0.070 |  |  | *Aggressive CD symptoms* | 1.125^*^ | 1.059 – 1.195 | 0.031 |
|  | *Non-aggressive CD symptoms* | 0.238^*^ | 0.092 – 0.389 | 0.075 |  |  | *Non-aggressive CD symptoms* | 1.016 | 0.951 – 1.086 | 0.034 |
|  | *Aggressive CD symptoms* | 0.259^*^ | 0.069 – 0.455 | 0.098 |  |  | *Aggressive CD symptoms* | 1.042 | 0.848 – 1.299 | 1.110 |
|  | *Non-aggressive CD symptoms* | 0.303^*^ | 0.098 – 0.502 | 0.103 |  |  | *Non-aggressive CD symptoms* | 1.062 | 0.858 – 1.312 | 1.120 |
|  | *Aggressive × non-aggressive symptoms* | -0.084 | -0.227 – 0.055 | 0.071 |  |  | *Aggressive × non-aggressive symptoms* | 1.155 | 0.931 – 1.422 | 1.110 |
|  | *Block design* | 0.064 | -0.082 – 0.206 | 0.073 |  |  | *Block design* | 1.065 | 1.000 – 1.135 | 0.032 |
|  | *Vocabulary* | -0.039 | -0.186 – 0.105 | 0.074 |  |  | *Vocabulary* | 0.996 | 0.933 – 1.063 | 0.033 |
|  | *Block design* | 0.060 | -0.124 – 0.235 | 0.093 |  |  | *Block design* | 0.923 | 0.739 – 1.150 | 1.120 |
|  | *Vocabulary* | -0.033 | -0.312 – 0.200 | 0.129 |  |  | *Vocabulary* | 1.010 | 0.761 – 1.330 | 1.150 |
|  | *Block design × Vocabulary* | -0.025 | -0.172 – 0.106 | 0.074 |  |  | *Block design × Vocabulary* | 0.984 | 0.926 – 1.040 | 1.030 |
|  | *Vocabulary* | 0.247 | -0.112 – 0.609 | 0.185 |  |  | *Vocabulary* | 0.999 | 0.606 – 1.620 | 1.280 |
|  | *Maternal ASPD* | 0.115 | -0.192 – 0.409 | 0.155 |  |  | *Maternal ASPD* | 1.245 | 0.838 – 1.880 | 1.230 |
|  | *Vocabulary × Maternal ASPD* | -0.087 | -0.338 – 0.154 | 0.126 |  |  | *Vocabulary × Maternal ASPD* | 1.017 | 0.901 – 1.140 | 1.060 |
| **Arrest after 18^th^ birthday** | | ***e^β^*** | **95% CI** | ***SE*** |  | **Recent legal involvement** | | ***e^β^*** | **95% CI** | ***SE*** |
|  | *Aggressive CD symptoms* | 1.182 | 0.950 – 1.471 | 0.112 |  |  | *Aggressive CD symptoms* | 1.071 | 0.872 – 1.328 | 0.107 |
|  | *Non-aggressive CD symptoms* | 1.032 | 0.819 – 1.300 | 0.117 |  |  | *Non-aggressive CD symptoms* | 1.059 | 0.846 – 1.313 | 0.112 |
|  | *Aggressive CD symptoms* | 1.220 | 0.583 – 2.725 | 1.480 |  |  | *Aggressive CD symptoms* | 1.295 | 0.964 – 1.760 | 1.170 |
|  | *Non-aggressive CD symptoms* | 1.097 | 0.854 – 1.426 | 1.140 |  |  | *Non-aggressive CD symptoms* | 0.858 | 0.614 – 1.180 | 1.180 |
|  | *Aggressive × non-aggressive symptoms* | 1.158 | 0.908 – 1.485 | 1.130 |  |  | *Aggressive × non-aggressive symptoms* | 1.177 | 0.947 – 1.460 | 1.120 |
|  | *Block design* | 0.871 | 0.702 – 1.075 | 0.108 |  |  | *Block design* | 0.925 | 0.743 – 1.146 | 0.109 |
|  | *Vocabulary* | 0.760^*^ | 0.607 – 0.958 | 0.117 |  |  | *Vocabulary* | 0.777^*^ | 0.628 – 0.957 | 0.108 |
|  | *Block design* | 0.317^*^ | 0.115 – 0.837 | 1.670 |  |  | *Block design* | 0.861 | 0.699 – 1.051 | 1.110 |
|  | *Vocabulary* | 0.422 | 0.133 – 1.195 | 1.740 |  |  | *Vocabulary* | 0.750^*^ | 0.598 – 0.934 | 1.120 |
|  | *Block design × Vocabulary* | 0.911 | 0.713 – 1.167 | 1.130 |  |  | *Block design × Vocabulary* | 1.085 | 0.898 – 1.314 | 1.100 |
|  | *Vocabulary* | 0.763 | 0.408 – 1.420 | 1.380 |  |  | *Vocabulary* | 0.728 | 0.467 – 1.128 | 1.250 |
|  | *Maternal ASPD* | 0.964 | 0.557 – 1.720 | 1.330 |  |  | *Maternal ASPD* | 1.142 | 0.777 – 1.695 | 1.220 |
|  | *Vocabulary × Maternal ASPD* | 0.902 | 0.516 – 1.560 | 1.330 |  |  | *Vocabulary × Maternal ASPD* | 0.772 | 0.490 – 1.202 | 1.260 |

*Note.* Each augmented model included every component of the corresponding compact model, yet was estimated separately from the other corresponding augmented models to maximize sample size. Each augmented model row contains parameter estimates from generalized linear mixed models regressing the outcome variable on that particular predictor after controlling for demographic factors, sample, and baseline conduct disorder. That is, each estimate was performed in the context of a separate model. See Table 3 for further details. (standardized with respect to the predictor but not the outcome)

^*^95% credibility interval doesn’t cover zero or one for linear and exponentiated regression weights, respectively,

Figure A. Distributions of observed and imputed outcomes: past year and lifetime ASPD symptoms


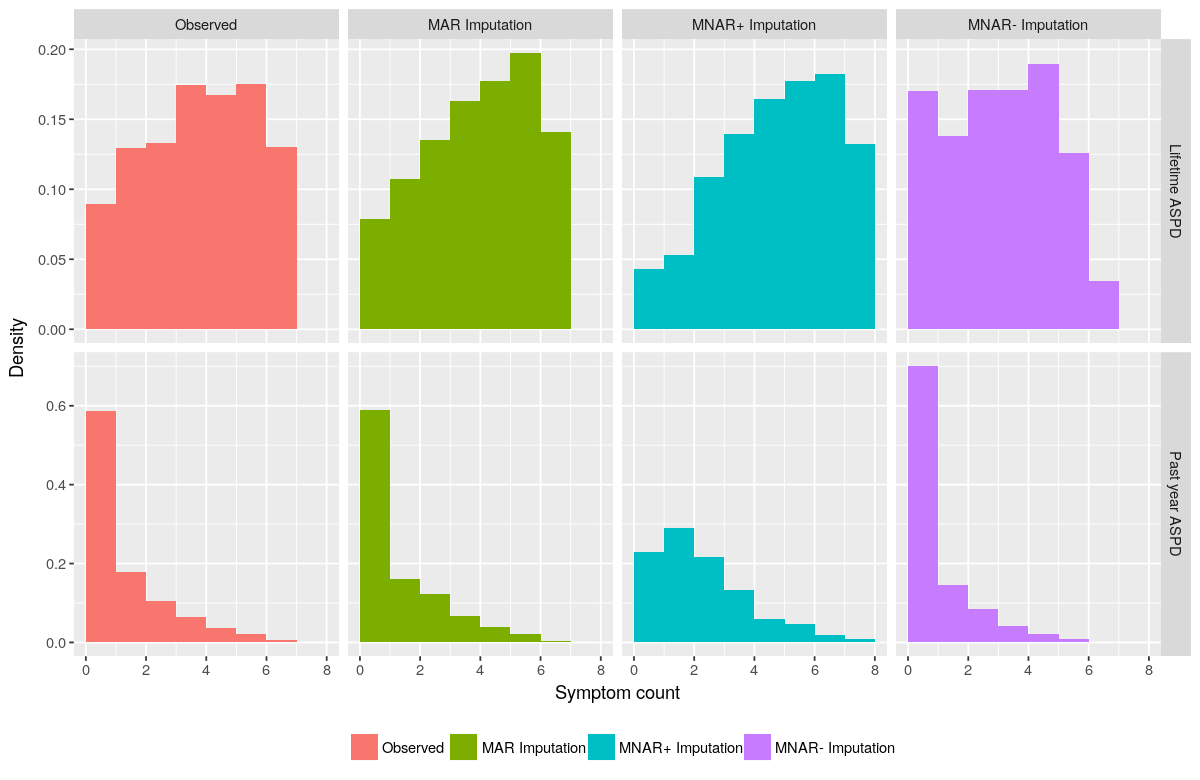


*Note. MAR* replicate data sets were constructed via multivariate imputation by chain equations using predictive mean matching for lifetime ASPD and an augmented Poisson general linear models for past year ASPD. This procedure is valid under the assumption that attrition and outcome severity are conditionally independent given the covariates. *MAR* replicates were further manipulated by adding structured random offsets to each outcome to simulate different non-random attrition regimes. *NMAR+* replicates result from simulating a positive association between attrition and outcome severity, and *NMAR-* replicates result from simulating a negative association between attrition and outcome severity. This was accomplished by augmenting attrited participants’ *MAR*-imputed symptom counts with positive or negative binomial (n = 2, p = .5) random offsets corresponding to one average symptom differences (SD = .707) under the *NMAR+* and *NMAR-* regimes, respectively.

Figure B. Distributions of observed and imputed outcomes: arrest after 18^th^ birthday and recent legal involvement


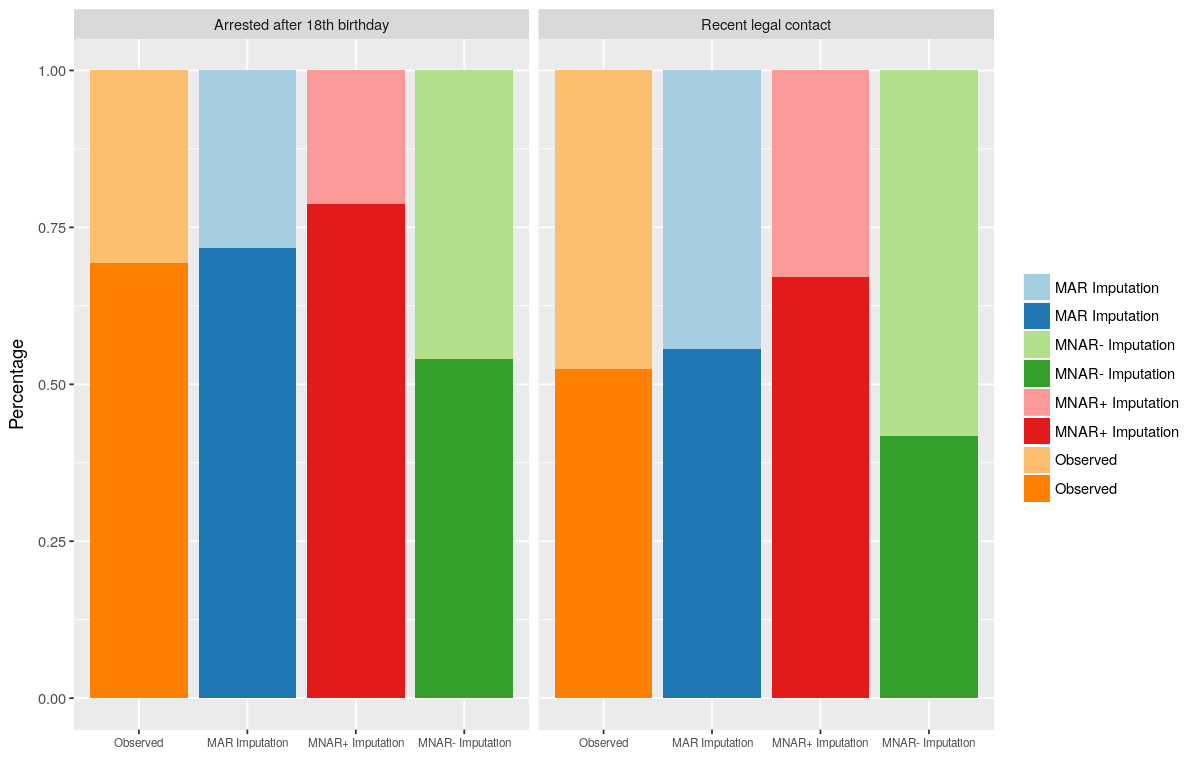


*Note.* Replicate datasets were constructed via multivariate imputation by chain equations using augmented Bernoulli general linear models. *MAR* analyses were performed on these data, and are valid under the assumption that attrition and outcome severity are conditionally independent given the covariates. *MAR* replicates were further manipulated by adding structured random offsets to each outcome to simulate different non-random attrition regimes. *NMAR+* replicates result from simulating a positive association between attrition and outcome severity, and *NMAR-* replicates result from simulating a negative association between attrition and outcome severity. This was accomplished by augmenting attrited participants’ *MAR*-imputed symptom counts with positive or negative Bernoulli (p = .25) random offsets. Attrited participants who were imputed as not having experienced arrest under the *MAR* regime had a 25% chance being imputed as arrested under the *NMAR+* regime. Likewise, attrited participants who were imputed as having experienced recent legal involvement under the *MAR* regime had a 25% chance being imputed as not experiencing recent legal involvement under the *NMAR-* regime.

Figure C. Predictor slopes by sample: past year and lifetime ASPD symptoms

*
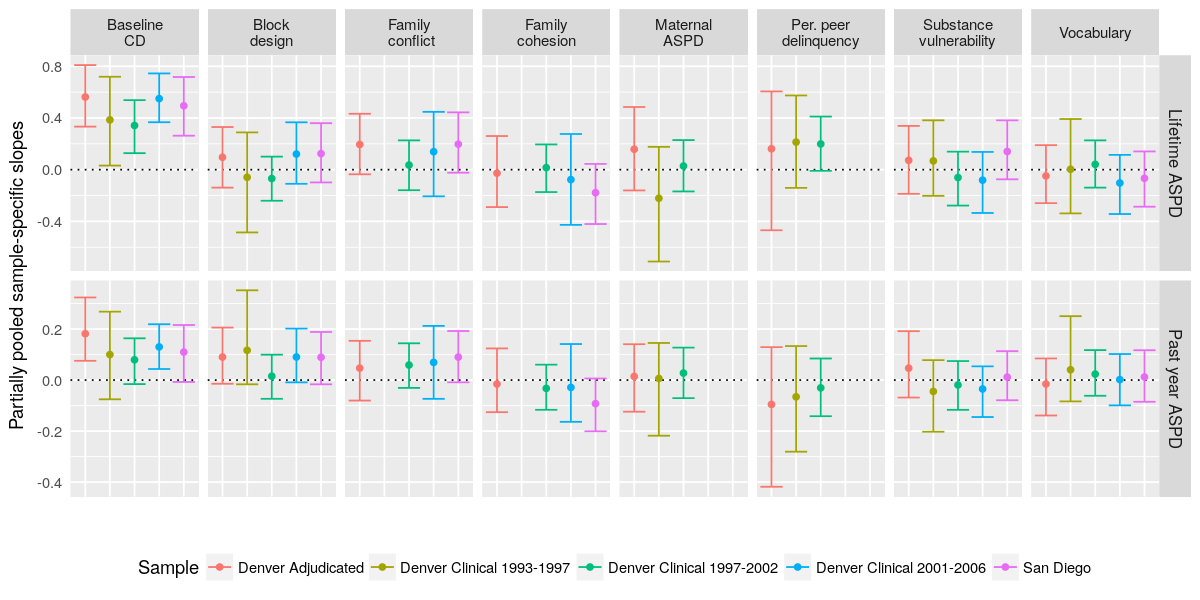
*

*Note*: Partially pooled slope estimates for each psychosocial predictor across samples. For each predictor, the equivalent model presented in Table 3 was augmented with a random slope for the predictor across samples. Additionally, random slopes for baseline CD were included in each model. These slopes are similar to what would be obtained by running each model in a particular sample, except effects of sex, age, and ethnicity slopes are constrained across samples and slopes for psychosocial predictors display shrinkage toward the mean slope across samples. For the sake of comparability across linear and nonlinear models, all coefficients are un-exponentiated.

Figure D. Predictor slopes by sample: arrest after 18th birthday and recent legal involvement


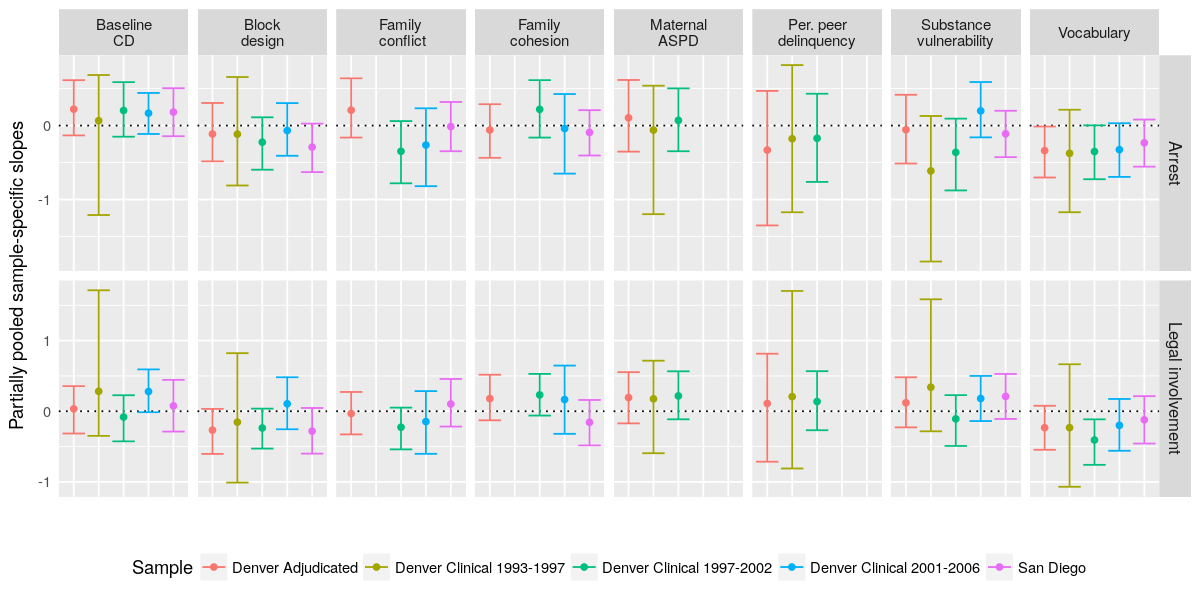


*Note*: Partially pooled slope estimates for each psychosocial predictor across samples. For each predictor, the equivalent model presented in Table 4 was augmented with a random slope for the predictor across samples. Additionally, random slopes for baseline CD were included in each model. These slopes are similar to what would be obtained by running each model in a particular sample, except effects of sex, age, and ethnicity slopes are constrained across samples and slopes for psychosocial predictors display shrinkage toward the mean slope across samples. For the sake of comparability across linear and nonlinear models, all coefficients are un-exponentiated.
